# Ammonia oxidizers in the sea-surface microlayer of a coastal marine inlet

**DOI:** 10.1371/journal.pone.0202636

**Published:** 2018-08-20

**Authors:** Shu-Kuan Wong, Minoru Ijichi, Ryo Kaneko, Kazuhiro Kogure, Koji Hamasaki

**Affiliations:** 1 Laboratory of Marine Microbiology, Department of Marine Ecosystem Dynamics, Atmosphere and Ocean Research Institute, The University of Tokyo, Kashiwa, Chiba, Japan; 2 National Institute of Polar Research, Tachikawa, Tokyo, Japan; Universidade Federal do Rio de Janeiro, BRAZIL

## Abstract

Planktonic archaea are thought to play an important role in ammonia oxidation in marine environments. Data on the distribution, abundance, and diversity of ammonia oxidizers in the coastal sea-surface microlayer (SML) are lacking, despite previous reports of high abundance of *Thaumarchaeota* in the SML of estuaries and freshwater lakes. Here, we failed to detect the presence of ammonia-oxidizing bacteria in any of our samples taken from a semi-enclosed marine inlet in Japan. Therefore, we shifted our focus to examine the archaeal community composition as well as the *Thaumarchaeota* marine group I (MG-I) and ammonia monooxygenase subunit A (*amoA*) gene copy numbers and composition in the SML and corresponding underlying water (UW, 20 cm). *amoA* gene copy numbers obtained by quantitative PCR were consistent with the typical values observed in the surface waters of oceanic and coastal environments where nitrification activity has been detected, but the copy numbers were two- to three-fold less than those reported from the surface layers and UW of high mountain lakes. Both *amoA* and MG-I 16S rRNA gene copy numbers were significantly negatively correlated with chlorophyll-*a* and transparent exopolymer particle concentrations in the SML. Communities of archaea and ammonia-oxidizing archaea in SML samples collected during low wind conditions (≤5 m s^–1^) differed the most from those in UW samples, whereas the communities in SML samples collected during high wind conditions were similar to the UW communities. In the SML, low ratios of *amoA* to MG-I 16S rRNA genes were observed, implying that most of the SML *Thaumarchaeota* lacked *amoA*. To our knowledge, our results provide the first comparison of ammonia-oxidizing communities in the coastal SML with those in the UW.

## Introduction

Nitrification is an important process in the nitrogen cycle. The nitrification process involves a two-step biological conversion of ammonia to nitrate via nitrite. Ammonia oxidation—the first, and rate-limiting, step in nitrification—was once thought to be restricted to ammonia-oxidizing bacteria (AOB) belonging to the phylum *Proteobacteria* [1,2]. The discovery of the putative archaeal ammonia monooxygenase subunit A (*amoA*) gene [3] and the subsequent isolation of *Nitrosopumilus maritimus*, a member of the ammonia-oxidizing archaea (AOA) [4] that belongs to the phylum *Thaumarchaeota*, have provided new insights into the distribution of AOA and their roles in the nitrogen cycle. In marine environments, AOA are considered to be more abundant, and to play a more important role in ammonia oxidation, than AOB [5].

The sea-surface microlayer (SML) is a thin surface film located at the interfacial point between the sea surface and the atmosphere. SML archaeal communities differ from those in the underlying water, and SML-exclusive archaeal groups have been detected [6,7]. In high mountain lakes, members from the phylum *Thaumarchaeota* dominate the surface-layer archaeal community, whereas members of the phylum *Euryarchaeota* were more abundant in the subsurface waters [8–10]. Despite intensive studies of the ubiquitous distribution of AOA in marine environments, in the case of the SML, AOA community structure and abundance have been described only in a high mountain lake [11]. The presence of Marine Group-I *Thaumarchaeota* (MG-I) and *amoA* genes in the SML indicates the potential for ammonia oxidation in this layer. Furthermore, ammonia, the substrate needed for ammonia oxidation, is known to accumulate in the SML [12,13]. However, to date, no data are available on the ammonia-oxidizing communities in coastal and oceanic SML environments. Much less is known about the ammonia-oxidizing community in the SML than about ammonia oxidizers in other aquatic systems.

Here, we describe the archaeal community composition, and specifically the composition of *Thaumarchaeota* MG-I, as well as, MG-I, AOA and AOB gene copy numbers and composition, in the SML and the corresponding underlying water (UW, 20 cm depth) of a semi-enclosed marine inlet in Japan. Our findings regarding the ammonia-oxidizing communities in the SML and UW of this coastal inlet should fill the fundamental gaps in our knowledge of these globally important functional groups of microbes in one of the most under-studied areas of the marine environment.

## Materials and methods

### Study site

**Fig 1:**
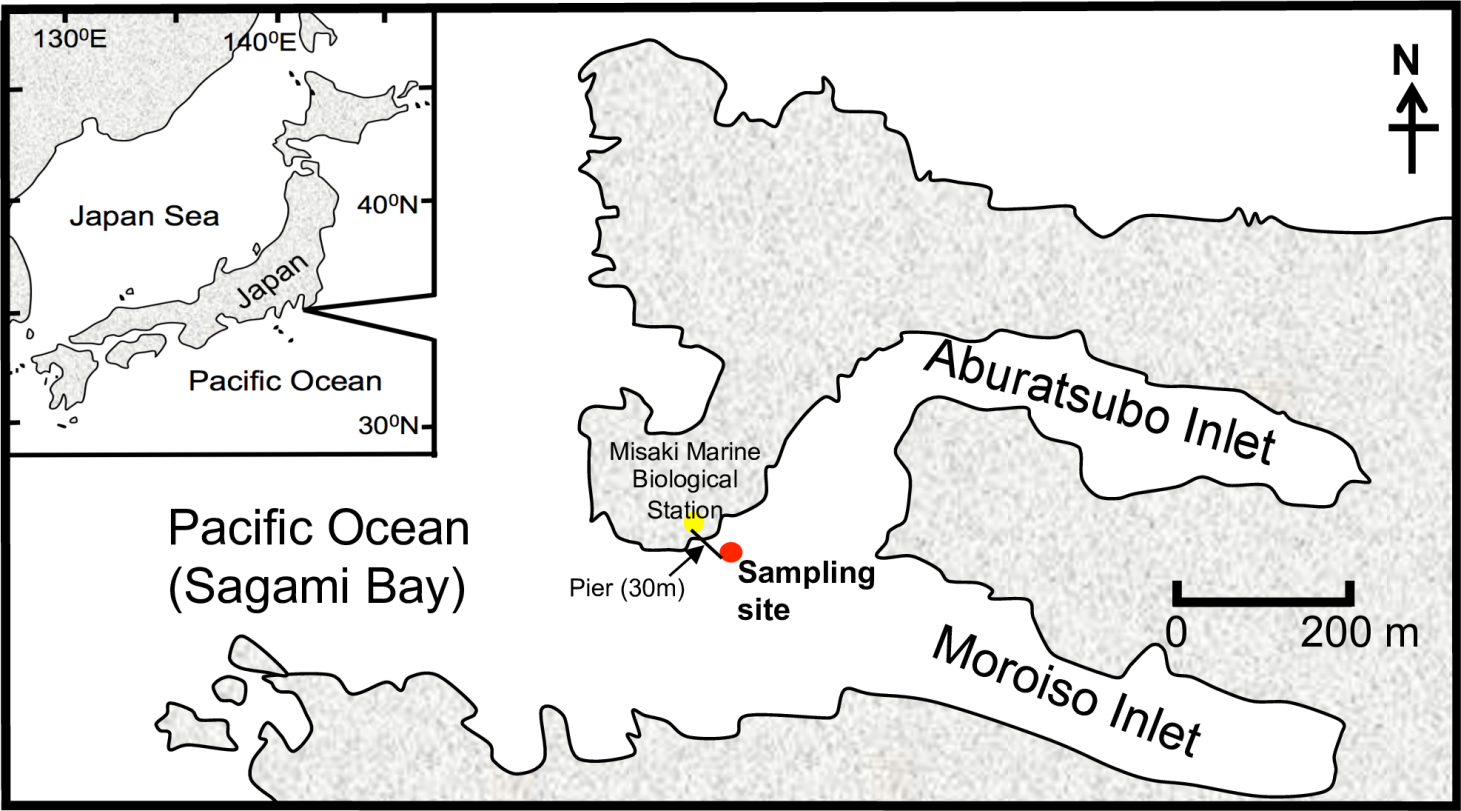
Sampling location. The location of the Misaki Marine Biological Station is indicated by the yellow dot, and the sampling point is indicated by the red dot.

Water samples from Aburatsubo Inlet, in the town of Misaki, in Kanagawa Prefecture, Japan, were obtained from the pier of Misaki Marine Biological Station in September and December 2012 at high tide to avoid resuspended bottom sediments. The sampling site (35°09.5′N, 139°36.5′E) was located approximately 30 m from land, with a maximum water depth of about 5 m (Fig 1). The semi-enclosed inlet is located in a temperate coastal area adjacent to Sagami Bay, which connects to the Pacific Ocean. The topography of the inlet was ideal for SML sampling, as it reduced the effect of oceanic waves from Sagami Bay.

### SML and UW sampling

SML water samples were collected by rotating a Plexiglas drum at a rate of 6 rotations min^–1^. The rotating drum sampler is widely used to sample large volumes of water from the SML of various environments, including the open ocean and coastal waters [14–16]. Water adhering to the drum was scraped off by using a Teflon scraper into a sterile collection bottle. To condition the drum surface, the first 1 L of SML water sample collected was discarded. Duplicate water samples from the SML were collected by using the drum sampler, and these duplicate samples were pooled for further filtration and analysis. The thickness of the SML sampled by the drum sampler was determined by dividing the volume of water sampled by the surface area of the sampler and the number of drum rotations. The thickness of the sampled SML was 36 ± 4 μm. At the same time as the drum samples were taken, a sterile bottle was submerged to a depth of 20 ± 5 cm to collect UW samples. To prevent contamination with SML water, the cap of the sterile bottle was opened and closed at a depth of 20 cm.

In total, 12 samples (six pooled samples from the SML and six samples from the UW) were collected. The samples were labeled, in sequence, according to the sampling month (September: S; December: W), sample number (e.g. 1, 2) and depth (SML samples collected using drum sampler: D; UW: U). A set of paired samples (SML and UW) was collected daily over 2 or 3 consecutive days from the same sampling point. The temperature and salinity of surface water samples were monitored by using a YSI Model 85 handheld meter (YSI Incorporated, Yellow Springs, USA). The development of a distinct microbial community in the SML requires relatively calm surface conditions and low wind speed [17]. Therefore, the wind speed during sampling was constantly recorded by using a handheld anemometer (GA-06, Be-s Co. Ltd, Osaka, Japan).

### Chlorophyll-*a* and transparent exopolymer particles as proxies for SML formation

Concentrations of transparent exopolymer particles (TEP) and chlorophyll-*a* (Chl-*a*) were measured as proxies of organic matter concentrations in the SML. TEP concentrate in the SML and are a major component of it [18]. TEP concentration was quantified as previously described [19,20]. Triplicate 10- to 20-mL subsamples for TEP quantification were filtered onto 0.4-μm-pore-size polycarbonate membrane filters (Isopore HTTP, Millipore, Tokyo, Japan), stained with 500 μL of 0.02% 8GX alcian blue solution (Sigma-Aldrich, Tokyo, Japan) in 0.06% acetic acid (pH 2.5), extracted in 80% sulfuric acid for 2 h, and then measured spectrophotometrically.

For Chl-*a* analysis, 50 mL each of duplicate subsamples were filtered onto 25-mm GF/F filters (GE Healthcare, Tokyo, Japan) extracted with N,N-dimethylformamide, and stored at −20°C until further analysis [21]. Chl-*a* concentrations were measured fluorometrically with a model 10-AU Field and Laboratory Fluorometer (Turner Designs, San Jose, USA).

The enrichment factors (EF) of TEP and Chl-*a* in the SML were calculated as the ratio of the concentration in the SML to that in the UW. These parameters were considered enriched in the SML when the EF value exceeded a ratio of 1.0 [22].

### Nucleic acid extraction

Approximately 1 L of water samples collected from the SML and UW were filtered onto 0.22-μm-pore-size Sterivex GP filter units (Millipore). The filters were immediately stored at −80°C until further analysis. DNA extraction was performed with a ChargeSwitch Forensic DNA Purification Kit (Invitrogen, Tokyo, Japan) after bead beating with zirconia beads (FastGene, Tokyo, Japan) at 5,000 rpm for 30 s. Each filter was extracted twice to maximize the DNA yield.

### Archaeal 16S rRNA gene pyrosequencing and data processing

The V1 to V3 hyper-variable regions of the archaeal 16S rRNA gene were amplified by using the forward primer A20F [23] and reverse primer 519R [24]. PCRs for each sample were performed in triplicate with 20 μL of mixture consisting of 6 μL of 10-fold diluted template DNA (5 to 8 ng μL^–1^), 0.13 μM of each primer, 0.2 mM of each dNTP, 2 μL of 10× *Ex Taq* Buffer (Takara, Shiga, Japan), and 0.5 U of *Ex Taq* HS (Takara). Touchdown PCR was performed for a total of 35 cycles under the following conditions: initial denaturation at 94°C for 3 min, 10 cycles of denaturation at 98°C for 10 s, annealing at 68 to 59°C for 30 s, elongation at 72°C for 45 s; followed by 25 cycles of denaturation at 98°C for 10 s, annealing at 58°C for 30 s, elongation at 72°C for 45 s; and final elongation at 72°C for 10 min. The prepared PCR products were purified with an AMPure system (Beckman Coulter, Brea, USA) and sequenced at the Atmosphere and Ocean Research Institute, Chiba, on a Roche 454 GS Junior platform (Roche 454 Life Science, Brandford, USA) in accordance with the manufacturer’s instructions.

After the sequencing, the open-source software mothur 1.35.1 [25,26] was used for subsequent analysis in accordance with the 454 standard operating procedure (http://www.mothur.org/wiki/454_SOP; accessed on 23 March 2015). Reads for downstream sequence processing were retained if they had a minimum quality score of 30, and representative sequences were assigned to operational taxonomic units (OTUs) by using the furthest-neighbor clustering algorithm based on 97% identity.

### Detection of ammonia-oxidizing bacteria

The betaproteobacterial and gammaproteobacterial *amoA* gene fragments were amplified by using the amoAr-New/amoA-2R [27] and amoA3F/amoB-4R [28] primer sets, respectively. PCR for each sample was performed in triplicate with 20 μL of reaction mixture consisting of 3 μL of 10-fold-diluted template DNA (5 to 8 ng μL^–1^), 0.2 μM of each primer, 0.2 μM of each dNTP, 2 μL of 10× *Ex Taq* Buffer (Takara), and 0.5 U of *Ex Taq* HS Polymerase (Takara). Thermal cycling was performed under the following conditions: initial denaturation at 95°C for 3 min, 40 cycles of amplification at 94°C for 30 s, annealing at 47°C (amoAr-New/amoA-2R) and 48°C (amoA3F/amoB-4R) for 1 min, elongation at 72°C for 40 s, and a final elongation at 72°C for 10 min. As the amplification of AOB *amoA* from all samples was unsuccessful, no further analysis of AOB was done.

### Detection and quantification of ammonia-oxidizing archaea

The archaeal *amoA* gene fragments were quantified by using the Arch-*amoA*-for /Arch-*amoA*-rev primer set [5], whereas MG-I 16S rRNA gene fragments were quantified by using the GI-751F/GI-956R primer set [29] in triplicate by quantitative PCR (qPCR). Serial dilutions of known plasmid numbers were used to generate an external standard curve for qPCR analysis [*amoA*: kt162 plasmid, GenBank accession number: AB592102; and MG-I 16S rRNA gene: kt779 plasmid, GenBank accession number: AB919347]. qPCR was performed in reaction mixtures (20 μL) that contained 3 μL of 10-fold-diluted template DNA (5 to 8 ng μL^–1^), 10 μL of 2× SYBR *Premix Ex Taq* with Tli RNase H (Takara), and 0.2 μM final concentration of each primer. Assays for both primer sets were performed by using a Roche Light Cycler 480 II Real-Time PCR system (Roche, Sussex, UK) with the following thermal cycling conditions: initial denaturation at 95°C for 30 s; and 45 cycles of amplification at 95°C for 5 s, annealing at 58°C for 30 s, and elongation at 72°C for 30 s, with a detection step at the end of each cycle. After amplification, melting curve analyses were performed at 95°C for 5 s, followed by a temperature gradient of 0.11°C s^−1^ from 65 to 95°C. The qPCR detection efficiency was 95.1% for MG-I 16S rRNA genes and 90.0% for *amoA* genes.

### Construction of an archaeal *amoA* clone library and clone library sequence analysis

An approximately 256-bp fragment of archaeal *amoA* was amplified by using the Arch-*amoA*-for/Arch-*amoA*-rev primer set. PCR was performed in triplicate using a 20-μL reaction mixture consisting of 3 μL of 10-fold-diluted template DNA (5 to 8 ng μL^–1^), 0.2 μM of each primer, 0.2 mM of each dNTP, 2 μL of 10× *Ex Taq* Buffer, and 0.5 U of *Ex Taq* HS (Takara). The PCR thermal cycling conditions consisted of initial denaturation at 95°C for 3 min; 40 cycles of amplification at 98°C for 10 s, annealing at 58°C for 30 s, and elongation at 72°C for 1 min; and final elongation at 72°C for 10 min. Amplified PCR products were pooled and purified by using a Qiaquick PCR Purification Kit (Qiagen KK, Tokyo, Japan). Cloning was performed with a TOPO TA Cloning Kit for Sequencing (Invitrogen) and *Escherichia coli* DH5-α competent cells (Takara). Positive clones with PCR products amplified through colony PCR were purified by using USB ExoSAP-IT product cleanup reagent (Affymetrix Japan, Tokyo, Japan) and sequenced by using an ABI Prism BigDye Terminator v3.1 Cycle Sequencing Kit (Applied Biosystems, Tokyo, Japan) on an ABI 3730xl platform (Applied Biosystems).

A total of 387 sequences obtained from the clone library were aligned by using MEGA 6.0 [30] against reference sequences retrieved from the GenBank database, and sequences with regions that were ambiguously aligned or with ambiguous amino acid sequences were removed. The remaining sequences were clustered into OTUs by using CD-HIT [31] on the basis of 95% nucleotide identity; this yielded 22 representative *amoA* OTUs. The 22 representative OTU sequences at 95% nucleotide identity (by CD-HIT) and the closest relative reference sequences from GenBank [32] were used to construct a neighbor-joining phylogenetic tree with the Tamura-Nei model [33] of nucleotide substitution and 1000 bootstrap replicates, implemented in MEGA 6.0.

### Statistical analysis

Samples from both samplings (September and December) were pooled according to their depth, SML or UW, for statistical analysis using R version 3.2.3 [34]. The correlation between *amoA* or MG-I 16S rRNA gene copy numbers and Chl-*a* and TEP concentrations in all pooled samples were calculated in R by using the non-parametric Spearman’s rank coefficient test. Differences in Chl-*a* and TEP concentrations between the SML and UW were tested by using the non-parametric Mann-Whitney U-test in R. Principal coordinate analysis (PcoA) of archaeal community structure based on weighted and unweighted UniFrac distances and of AOA community structure based on *amoA* gene clones was conducted in R by using the phyloseq package [35]. Phylogenetic trees used in the UniFrac analysis were generated using the clearcut function [36] in mothur 1.35.1 [25,26]. Differences between the SML and UW archaeal 16S rRNA gene communities and *amoA* clone libraries were tested by using weighted and unweighted UniFrac and analysis of molecular variance (AMOVA) functions in the mothur 1.35.1 platform according to the default parameters.

### Nucleotide sequence accession numbers

The *amoA* sequences reported here were deposited in GenBank under accession numbers KU216137 to KU216158. The archaeal 16S rRNA gene sequences obtained by pyrosequencing were deposited in the DNA Data Base of Japan Sequence Read Archive (http://trace.ddbj.nig.ac.jp/dra/index_e.html) under the accession number DRA004205.

## Results

### Environmental conditions and enrichment of organic matter in the SML

Sea-surface temperature in Aburatsubo Inlet peaks (at 25 to 26°C) with low salinity in August and September (summer), and the temperature reaches its lowest (15 to 16°C) with high salinity in December (winter) [37]. The temperature and salinity ranges obtained during our sampling in September (26.2 to 27.7°C; salinity, 31.7 to 32.4) and December (14.3 to 15.9°C, 34.1 to 34.2) corresponded to the summer and winter values previously reported by Yamaguchi [37]. Salinity in the Aburatsubo Inlet remained similar to that of the surrounding ocean, with slight seasonal changes; there is no known direct river input into the inlet [38]. Ammonium and phosphate concentrations in the semi-enclosed inlet are also higher in summer (September) than in winter (December) [38].

**Fig 2:**
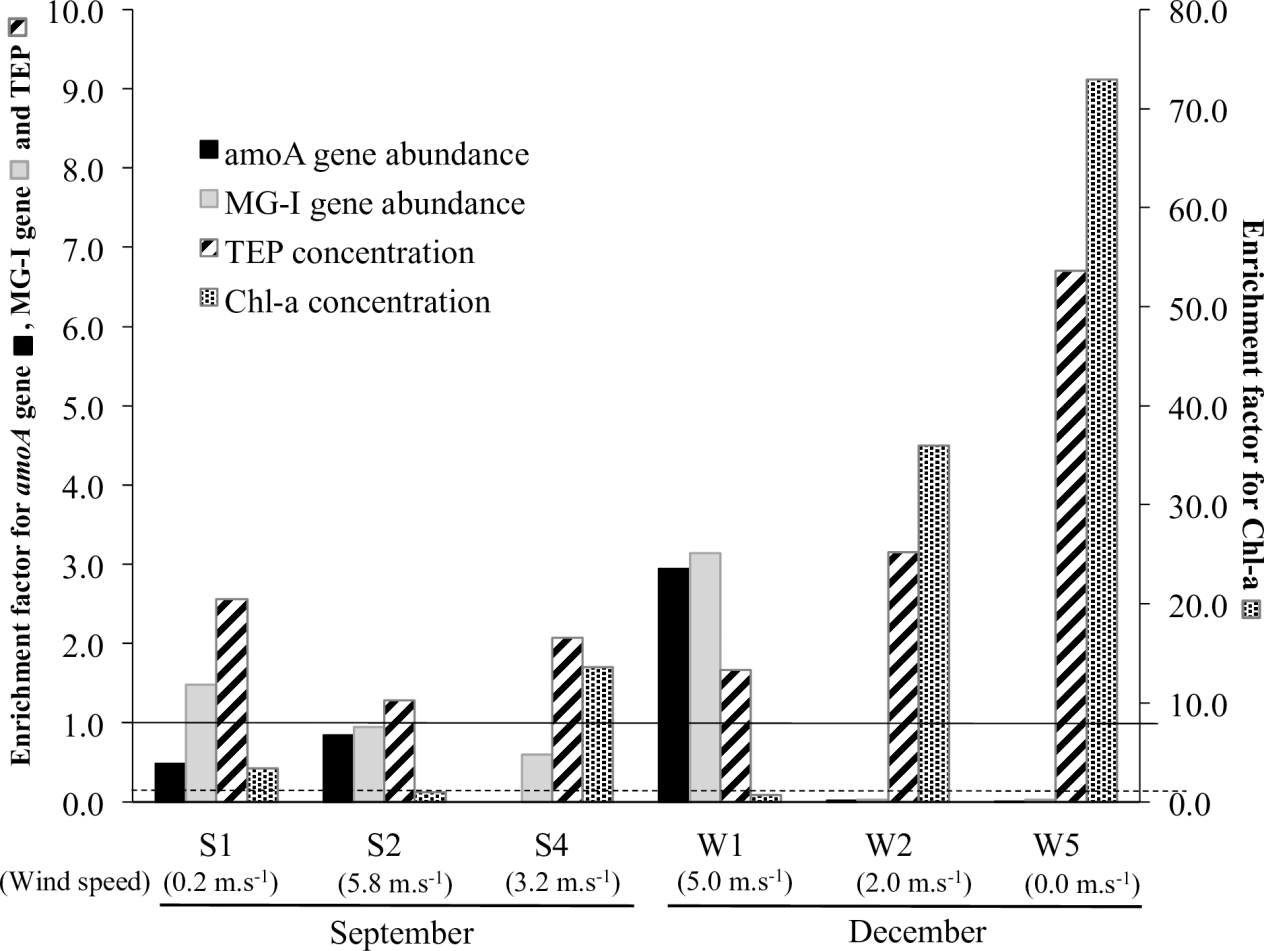
Enrichment factors of archaeal ammonia monooxygenase subunit A (*amoA*) gene copy numbers, archaeal Marine Group-I (MG-I) 16S rRNA gene copy numbers, transparent exopolymer particles (TEP) concentration, and chlorophyll-*a* (Chl-*a*) concentration in the sea-surface microlayer (SML) in samples collected in September (S) and December (W). Enrichment value of 1.0 is indicated by a solid line extending from the first *y*-axis in the case of *amoA* copy numbers, MG-I 16S rRNA gene copy numbers, and TEP concentrations and by a dashed line extending from the secondary *y*-axis in the case of Chl-*a* concentration. Enrichment >1.0 indicates enrichment in the SML.

EF ranged from 1.3 to 6.7 for TEP and 0.7 to 72.9 for Chl-*a* throughout the sampling periods in September and December (Fig 2). Chl-*a* and TEP concentrations were significantly positively correlated (*P* < 0.001; S1 Table). When the wind speed was low (≤5.0 m s^−1^), the EF for all Chl-*a* samples exceeded 1.0 (SML enriched) whereas the TEP was constantly enriched in the SML regardless of the wind speed (Fig 2). The Chl-*a* (Mann–Whitney U, *P* = 0.06) and TEP (Mann-Whitney U, *P* < 0.01) concentrations were also significantly higher in the SML than in the UW at times of low wind speed. The highest EF values for Chl-a and TEP were observed in December, when the wind speed was the lowest (Fig 2). At high wind speeds (≥5.0 m s^−1^), the EF values in the SML for Chl-a were lowest and the TEP enrichment in the SML was lower.

### Archaeal community structure

**Fig 3:**
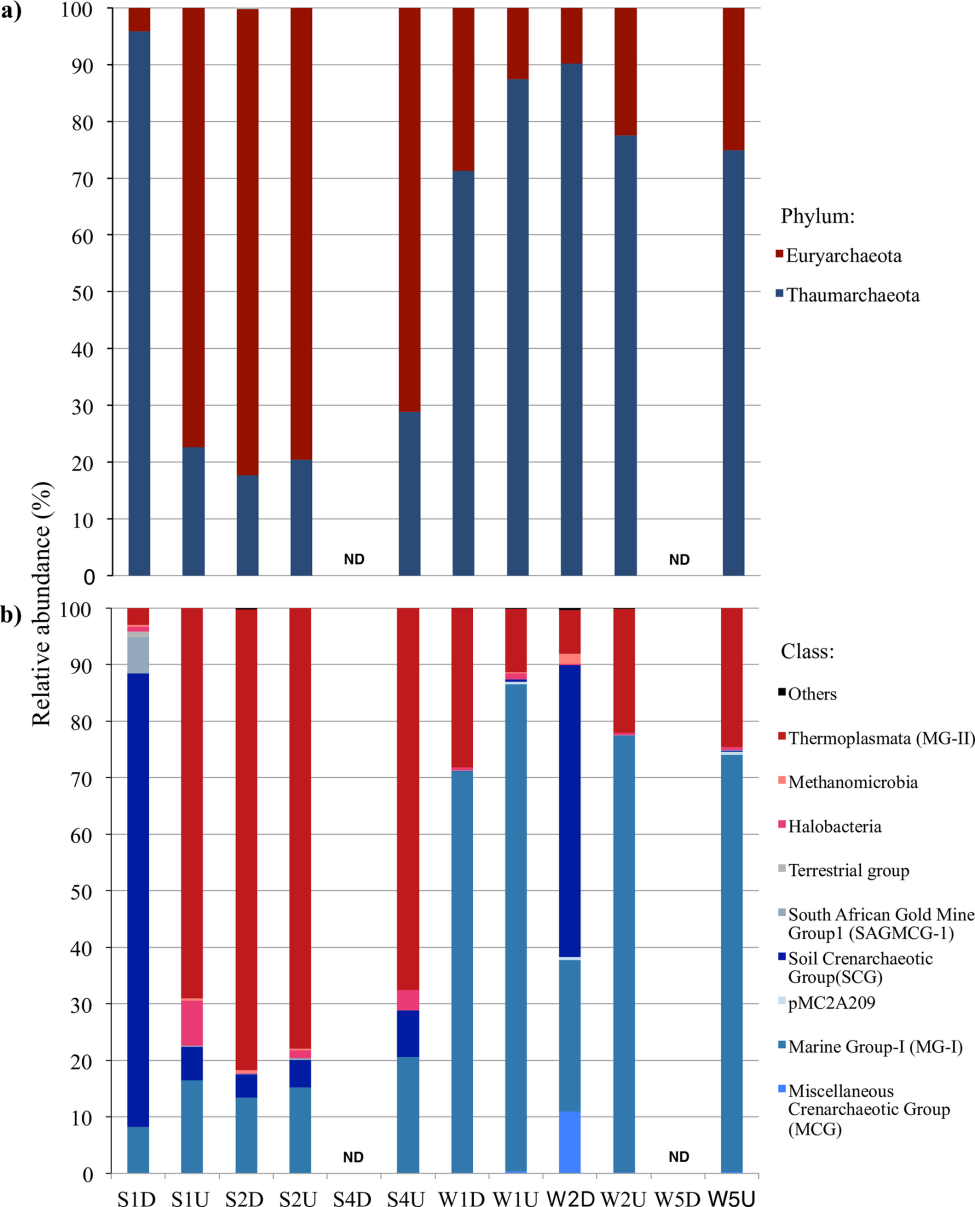
Relative abundances of archaeal taxonomic groups, obtained by using 454 pyrosequencing of the 16S rRNA gene, at the a) phylum and b) class levels for surface microlayer (D) and underlying water (U) samples obtained in September (S) and December (W). Abundances are presented in terms of percentage of total archaeal operational taxonomic units at a 97% identity level. “ND” indicates that the sample was not successfully amplified by using the A20F/519R primer set for 454 pyrosequencing.

The archaeal community from MG-II belonging to the phylum *Euryarchaeota* was more abundant in the SML and UW (except in sample S1D) in the September samples than in the December samples, with an average relative abundance of 77.6% ± 4.1% across both the SML and UW combined (Fig 3A). In December, the major archaeal phylum from SML and UW combined samples shifted to *Thaumarchaeota*, for which the relative abundance ranged from 71.3% to 90.0% (Fig 3A). Phylum *Thaumarchaeota* accounted for a large fraction of the archaeal community in the SML samples, with an average relative abundance of 68.8% (range: 17.7% to 95.9%) across all SML samples, excluding samples S4D and W5D, for which PCR amplification was unsuccessful (Fig 3A). The relative abundance of the Soil Crenarchaeota Group (SCG) (Fig 3B) was generally 50- to eight-fold higher in the SML samples from S1D and W2D than in the paired UW samples collected from the respective samplings, whereas the Terrestrial group was detected only in the S1D and W1D SML samples (Fig 2B). The PCoA ordination plot based on weighted UniFrac distances showed that the archaeal community composition and relative abundance in SML samples differed from those in the UW samples and SML samples that was collected during high wind speed (S1 Fig). All of the UW archaeal community was clustered closely together by sampling month. SML samples collected during times of high wind speed (≥5 m s^–1^) (S2D and W1D) were clustered close to the UW samples collected from the same respective samplings.

### Archaeal and bacterial *amoA* and MG-I 16S rRNA gene copy numbers

Interestingly, no beta- or gamma-proteobacterial *amoA* genes were detected in any of the SML or UW samples.

The average archaeal *amoA* copy numbers ranged from 0.04 to 11.89 × 10^5^ copies L^−1^ across all SML samples (with the exception of sample S4D, for which the value fell below the qPCR detection limit) and from 0.31 to 10.71 × 10^5^ copies L^−1^ across all UW samples. The copy numbers of the MG-I 16S rRNA gene across the SML samples ranged from 0.14 to 24.7 × 10^5^ copies L^−1^ and from 0.40 to 22.64 × 10^5^ copies L^−1^ across the UW samples (S2 Fig).

The copy numbers of *amoA* were significantly higher in UW than in the SML (Mann-Whitney U, *P* < 0.05) as evidenced by the enrichment values of *amoA*, which were depleted in the SML (EF = 0.34 ± 0.40), except in the case of sample W1 (EF = 2.9) (Fig 2). No significant differences were found between the SML and UW for MG-I 16S rRNA gene copy numbers from all samples from both samplings (Mann-Whitney U, *P* = 0.21). MG-I genes in the SML were enriched only in samples S1 and W1 (Fig 2). Significant positive correlations were found between *amoA* and MG-I 16S rRNA gene copy numbers (S1 Table). The ratio of *amoA* to MG-I 16S rRNA genes was, on average, 0.4 ± 0.2 in the SML and 0.7 ± 0.1 in the UW.

Both *amoA* and MG-I 16S rRNA gene copy numbers were negatively correlated with TEP (*amoA* gene number: *P* < 0.001; MG-I 16S rRNA gene number: *P* < 0.01) and Chl-*a* concentrations (*amoA* gene number: *P* < 0.001; MG-I 16S rRNA gene number: *P* < 0.001; S1 Table). In two samples from the SML (S4D and W5D) they either were present in very low abundance or were undetectable using qPCR primer sets targeting the archaeal *amoA* and MG-I 16S rRNA genes (S2 Fig). Furthermore, S4D and W5D samples were also unsuccessfully amplified using the current archaeal 16S rRNA gene primer set for pyrosequencing (A20F/519R; Fig 3). The patterns of low gene copy numbers or unsuccessful PCR amplifications from the two samples coincided with the highest peaks of both Chl-*a* and TEP in the SML during the respective samplings (S2 Fig).

### Community structure and diversity of AOA

**Fig 4:**
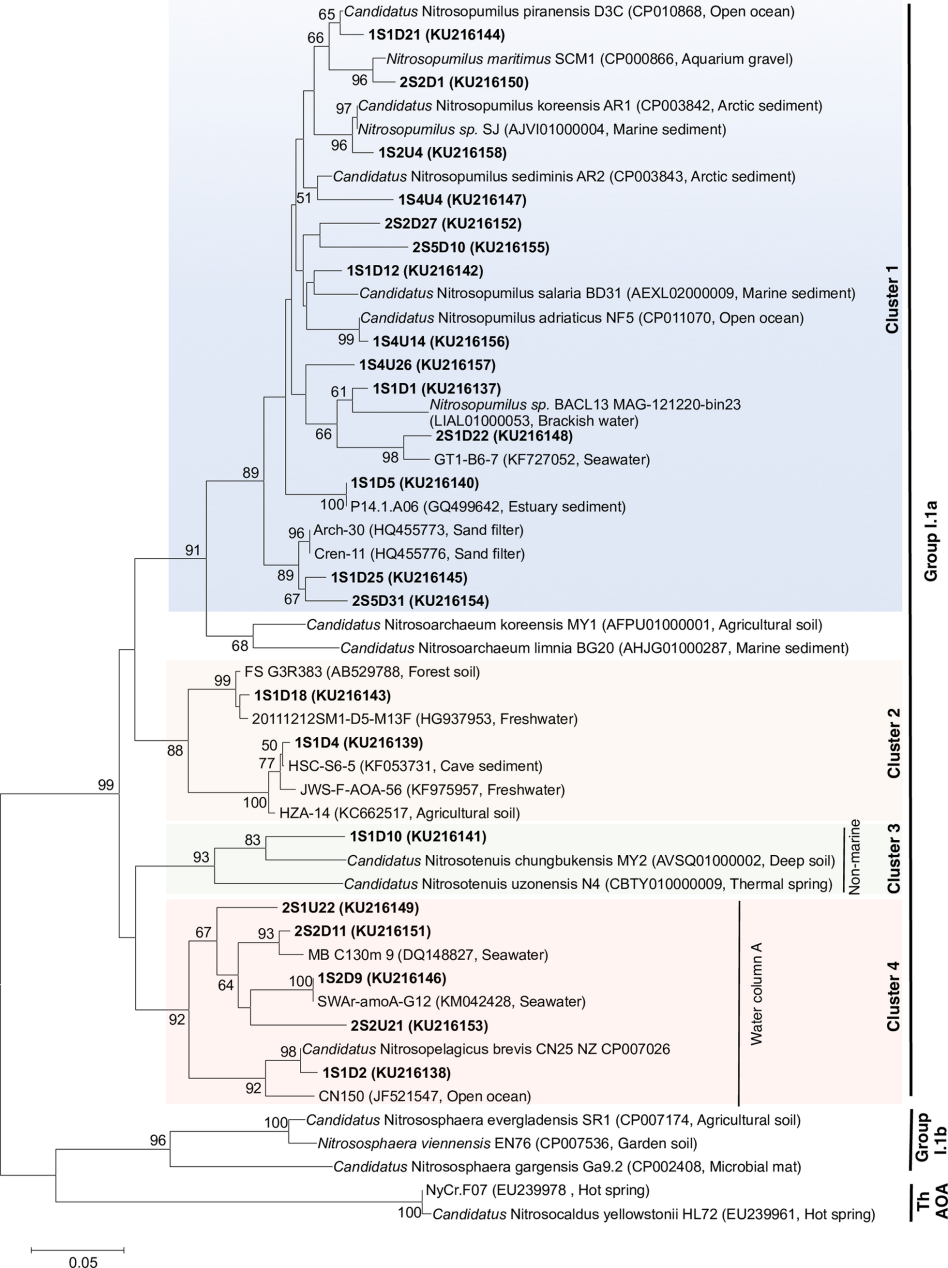
Consensus neighbor-joining phylogenetic tree of archaeal ammonia monooxygenase subunit A (*amoA*) gene nucleotide sequences based on Tamura-Nei distances. The tree contains 22 representative operational taxonomic units (OTUs) from this study (in bold) obtained at a cutoff value of 0.05% by using the CD-HIT program. Bootstrap values (>50%) are indicated at the nodes of the tree. Accession numbers for the representative OTUs and reference sequences are indicated in parentheses. Scale bar represents 0.05 amino acid substitutions per site.

**Fig 5:**
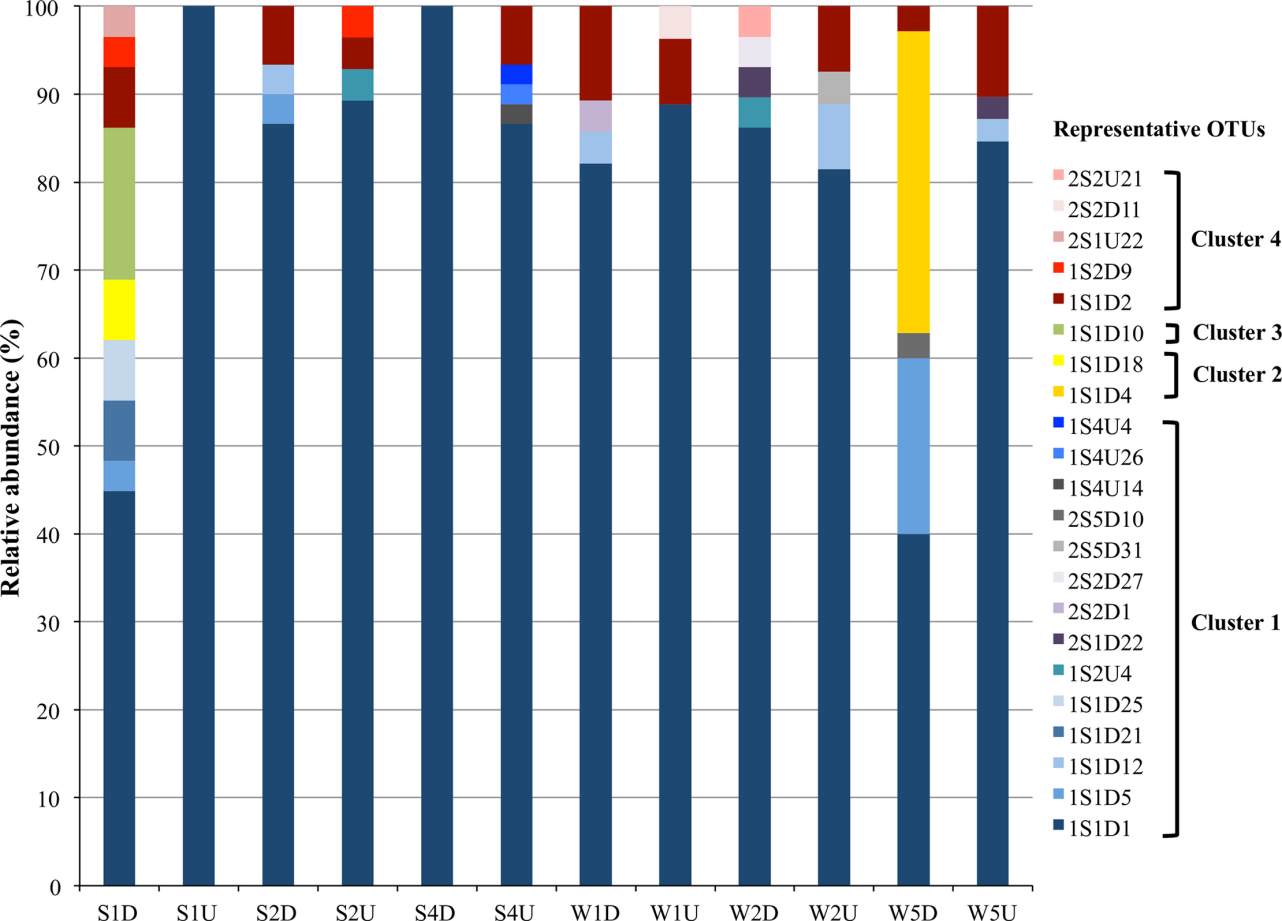
Relative abundances of archaeal ammonia monooxygenase subunit A (*amoA*) gene clones from surface microlayer (D) and underlying water (U) samples collected in September (S) and December (W). Clones were grouped into four different clusters based on representative operational taxonomic units with 95% sequence identity, obtained by using the CD-HIT program.

Four major clusters were detected in the SML and UW samples obtained in September and December; 329 of the clone sequences belonged phylogenetically to Cluster 1 (Fig 4), which consisted mainly of sequences that were closely related to the ammonia-oxidizing archaeon *Nitrosopumilus maritimus*. A majority of the phylotypes obtained from the SML and UW were also ubiquitously distributed within Cluster 4, which consisted of sequences from Water Column Cluster A (WCA) or a surface cluster [29,39]. SML-specific phylotypes represented by 1S1D5, 1S1D25, and 2S5D31 from Cluster 1, as well as 1S1D18 and 1S1D4 from Cluster 2, were found (Figs 4 and 5). These phylotypes were closely related to other clones obtained from sand filters, soil environments, and sediment environments, but not to any closely described species (Fig 5). Clone 1S1D10 from Cluster 3, which was found only in S1D (Fig 5), was clustered close to *Candidatus* Nitrosotenuis chungbukensis and *Candidatus* Nitrosotenuis uzonensis, which have been obtained from soil [40] and thermal spring enrichment cultures [41], respectively (Figs 4 and 5).

**Fig 6:**
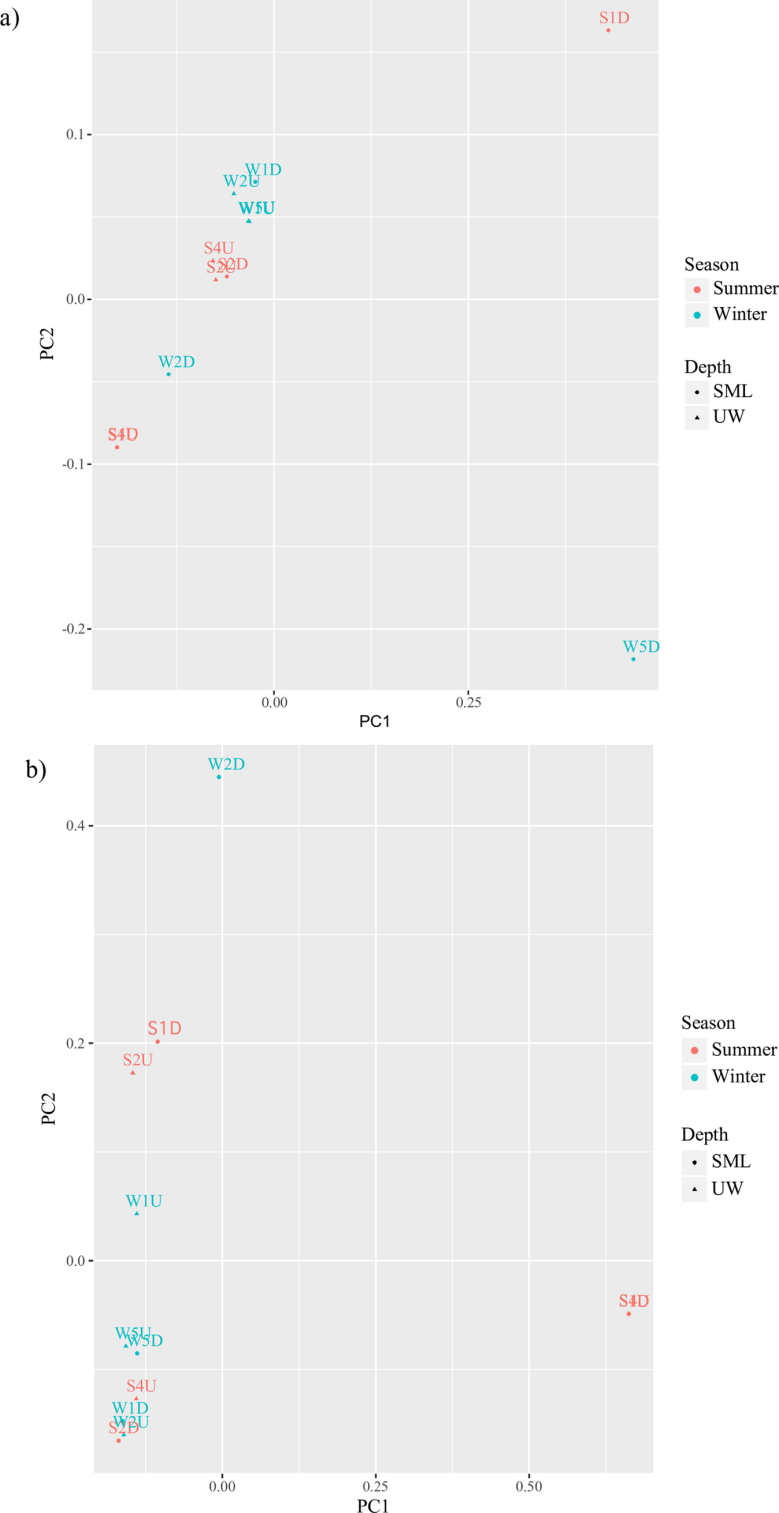
Principal coordinate analysis (PCoA) plots based on a) weighted and b) unweighted UniFrac distances of the *amoA* community structure from clone libraries.

AMOVA tests also showed significant differences between the SML and UW *amoA* structures (Fs = 3.02, *P* < 0.05). Additionally, both weighted (*P* < 0.001) and unweighted (*P* < 0.01) UniFrac showed that the AOA community structure and composition in the SML differed from those in the UW. All AOA communities from the UW were clustered closely in both September and December samplings, especially in the weighted UniFrac PCoA plot (Fig 6). Weighted and unweighted UniFrac analyses (Fig 6) revealed that AOA communities in the SML samples S2D and W1D, which were collected during high wind speed conditions, were also clustered close to the UW communities. Weighted UniFrac showed that the SML AOA communities from samples S1D, S4D, W2D, and W5D differed from their paired UW samples, whereas samples S1D, S4D, and W2D differed from their paired UW samples in the unweighted UniFrac plots.

## Discussion

### AOA and AOB gene copy numbers in the SML and UW

Our results provide insights into the gene copy numbers and diversity of AOA in the SML in coastal waters compared with those in the UW. In contrast to Zhang *et al*. [42], we did not amplify any beta- and gamma-proteobacterial *amoA* from any of our coastal SML and UW samples. Mincer *et al*. [29] and Beman *et al*. [43] also found that the gene copy number of AOB tended to show them as absent, or at lower numbers than AOA, in photic zones. Therefore, we surmise that ammonia oxidation in the SML and UW in our samples—if any—was performed mostly by AOA. The *amoA* and MG-I 16S rRNA gene datasets reported here are consistent with the typical values observed in the surface waters of oceanic and coastal environments [29,43,44] where nitrification activity has been detected [45]. However, they are two- to three-fold less than those reported in the surface layers and UW of high mountain lakes [8].

Our qPCR assays indicated that the *amoA* to MG-I 16S rRNA gene copy number ratio was within the reference ranges reported in oceanic regions [46–48]. However, the average *amoA* to MG-I 16S rRNA gene copy number ratio was lower for SML samples than for UW, suggesting that *amoA* genes in our samples were apparently less abundant than MG-I 16S rRNA genes. Currently, all *amoA* to MG-I 16S rRNA gene copy number ratios obtained from metagenomic [29] and genomic [49] sequences have been close to 1. However, lower ratios of *amoA* to MG-I 16S rRNA genes have also been reported previously [46,50]. The lower ratio obtained here could indicate that not all of the MG-I *Thaumarchaeota* possess *amoA*, or alternatively that the primer set we used was not able to amplify all of the *amoA* genes present in our samples. The primer set that we used has nucleotide mismatches, which may have led to spurious qPCR results and the underestimation of *amoA* copy numbers in the deep meso- and bathypelagic regions of the Pacific Ocean [47,51]. Despite the nucleotide mismatches, this primer set by Wuchter *et al*. [5] is able to detect *amoA* clades from both epipelagic and high-latitude regions [52,53].

Low ratios of *amoA* to MG-I 16S rRNA genes have also been attributed to a lack of *amoA* genes in the *Thaumarchaeota* [46,50,54]. Previous isotopic [55,56] and genomic [49,57] studies have shown that members of the phylum *Thaumarchaeota* are capable of assimilating organic matter to sustain their growth. The high concentrations of organic matter in surface waters—especially the SML—could theoretically attract heterotrophic *Thaumarchaeota*, thereby increasing the reliance of any *Thaumarchaeota* present in the SML on chemoorganoheterotrophy rather than chemolithoautotrophy.

### Archaeal and AOA community structures in the SML and UW

The *Thaumarchaeota* detected by amplicon sequencing and in the MG-I 16S rRNA gene qPCR results were more pronounced in the December SML and UW samples than in the samples collected during September. Decline of *Thaumarchaeota* during spring and summer [54,58,59] has been reported previously, suggesting the sensitivity of this group to photoinhibition or competition with bacteria [58]. Also, light is an important factor structuring the *Thaumarchaeota* population in the water column [29,47]. Although surface *Thaumarchaeota* may have developed pathways to help cope with UV-induced damage [60], it is still unclear whether ammonia oxidation is directly affected by photoinhibition.

MG-II from the phylum *Euryarchaeota* in the photic zone possesses the light-driven proton pump proteorhodopsin, providing an alternative pathway to support a photoheterotrophic lifestyle [61]. The higher relative abundance of MG-II *Euryarchaeota* in the SML and UW samples collected in September might indicate enhanced phototrophy in these surface waters in response to increased irradiance, as reported elsewhere [62,63]. A time-series study of planktonic archaea in the Santa Barbara Channel (Pacific Ocean off California, USA) has revealed that decreasing chlorophyll-*a* coincides with sporadic MG-II blooms in the surface waters [64].

Our comparisons between 16S rRNA gene archaeal community structure and *amoA* diversity and phylogeny showed that a portion of the archaeal communities in the coastal SML could be of sediment or terrestrial origin. In some of our samples—especially those collected when the wind speed was low and formation of the SML was thus maintained—phylotypes belonging to the SCG and Terrestrial groups were found in higher proportions in the SML than in the UW. AOA from soil environments (Clusters 2 and 3) were detected only in SML samples. SML-specific phylotypes closely related to AOA originating from soil, sediments, sand filters, and freshwater environments were present in our samples. This result is consistent with those of Stolle *et al*. [17], wherein a few bacterial OTUs closely related to sequences found in sediment environments were found specifically in the SML. The presence of these phylotypes could indicate passive transport via sediment resuspension, or intrusion of groundwater into the SML. It has also been speculated that the similarity of the chemical constituents in the SML to that of a solid substratum such as sediment could enable these newly introduced communities to be supported [65,66]. As these communities could have been passively transported into the water column and remained in the SML and UW for a period of time, further studies are needed to determine whether these SML-specific AOA communities are actively oxidizing ammonia and expressing *amoA* in the SML.

Differences in both total archaeal and AOA communities were observed between the SML and UW and were significantly correlated to the concentrations of organic matter (Chl-*a* and TEP). *amoA* and MG-I 16S rRNA gene copy numbers were especially low in our SML samples when Chl-*a* and TEP concentrations were high, and vice versa. Furthermore, the two SML samples that could not be amplified by the pyrosequencing primer sets had the lowest *amoA* and MG-I 16S rRNA gene copy numbers, indicating that archaeal abundance in the samples could have been very low. Interestingly, these samples contained the highest Chl-*a* and TEP concentrations in each sampling period. Negative correlations between *amoA* [62,67] or MG-I [8] gene copy numbers and Chl-*a* concentrations have been reported previously. One possible explanation is competition with phytoplankton [68]. It was recently discovered that competition with phytoplankton for ammonium could play a greater role than light intensity in regulating AOA cellular activity and nitrification rates [67]. However, there may be multiple factors regulating the *Thaumarchaeota* and the *amoA* composition and abundance: they could be affected by a wide array of environmental parameters, such as temperature [69,70], ammonia concentration [71], and even small changes in pH [72].

### Insights into archaeal dynamics in the SML

The presence of *amoA* and MG-I genes in the SML samples indicates the potential for ammonia oxidation in the top 1 mm of surface waters. However, the gene copy numbers of AOA tended to be low when the SML was well formed during low wind conditions. The lower ratio of *amoA* to MG-I 16S rRNA genes in the SML than in the UW could indicate that *Thaumarchaeota* present in the SML lack *amoA* and might rely on other substrates other than ammonia, such as organic matter, as an energy source. Additionally, the difference in archaeal and AOA community composition could reflect differences in nitrification between the SML and UW, as different AOA communities and clades possess different physiological and metabolic characteristics [45,52]. Because the physiology of AOA and the factors controlling their ammonia oxidation rates are still not well defined, future work should include the nitrification activity and gene transcript contents of AOA in the SML to help elucidate their role and adaptations to ammonia oxidation.

## Supporting information

S1 Table Non-parametric Spearman’s rank correlation coefficient test between the archaeal ammonia monooxygenase subunit A (*amoA*) gene and archaeal Marine Group-I (MG-I) 16S rRNA gene copy numbers relative to chlorophyll-*a* (Chl-*a*) and transparent exopolymer (TEP) concentrations. (DOCX)

S1 Fig Principal coordinate analysis (PCoA) plot based on weighted UniFrac distances in the archaeal 16S rRNA gene community structure, obtained by using 454 pyrosequencing. (PDF)

S2 Fig a) Copy numbers of archaeal ammonia monooxygenase subunit A (*amoA*) gene and archaeal Marine Group-I (MG-I) 16S rRNA gene; b) chlorophyll-*a* (Chl-*a*) and c) transparent exopolymer particles (TEP) concentrations from surface microlayer (D) and underlying water (U) samples collected in September (S) and December (W). * indicates that the concentration was below the qPCR detection limit and ** indicates that the sample was not amplified successfully using the A20F/519R primer set for 454 pyrosequencing. Error bars represent standard deviations of technical triplicates.
(PDF)

## References

[1] Teske A, Alm E, Regan JM, Toze S, Rittmann BE, Stahl DA. Evolutionary relationships among ammonia- and nitrite-oxidizing bacteria. J Bacteriol. 1994; 176(21): 6623–6630. 7961414 10.1128/jb.176.21.6623-6630.1994 PMC197018

[2] Ward BB, Capone DG, Zehr JP. What’s New in the Nitrogen Cycle? Oceanography. 2007; 20: 101–109.

[3] Venter JC, Remington K, Heidelberg JF, Halpern AL, Rusch D, Eisen JA, et al Environmental genome shotgun sequencing of the Sargasso Sea. Science. 2004; 304: 66–74. 10.1126/science.1093857 15001713

[4] Konneke M, Bernhard AE, de la Torre JR, Walker CB, Waterbury JB, Stahl, DA. Isolation of an autotrophic ammonia-oxidizing marine archaeon. Nature. 2005; 437: 543–546. 10.1038/nature03911 16177789

[5] Wuchter C, Abbas B, Coolen MJL, Herfort L, van Bleijswijk J, Timmers P, et al Archaeal nitrification in the ocean. Proc Natl Acad Sci USA. 2006; 103: 12317–12322. 10.1073/pnas.0600756103 16894176 PMC1533803

[6] Cunliffe M, Schafer H, Harrison E, Cleave S, Upstill-Goddard R, Murrell JC. Phylogenetic and functional gene analysis of the bacterial and archaeal communities associated with the surface microlayer of an estuary. ISME J. 2008; 2: 776–789. 10.1038/ismej.2008.28 18356822

[7] Cunliffe M, Harrison E, Salter M, Schaefer H, Upstill-Goddard RC, Murrell JC. Comparison and validation of sampling strategies for the molecular microbial analysis of surface microlayers. Aquat Microb Ecol. 2009; 57: 69–77.

[8] Auguet JC, Casamayor EO. A hotspot for cold crenarchaeota in the neuston of high mountain lakes. Environ Microbiol. 2008; 10: 1080–1086. 10.1111/j.1462-2920.2007.01498.x 18215160

[9] Auguet JC, Nomokonova N, Camarero L, Casamayor EO. Seasonal changes of freshwater ammonia-oxidizing archaeal assemblages and nitrogen species in oligotrophic Alpine lakes. Appl Environ Microbiol. 2011; 77(6): 1937–1945. 10.1128/AEM.01213-10 21239556 PMC3067326

[10] Villa-Costa M, Barberan A, Auguet JC, Sharma S, Moran MA, Casamayor EO. Bacterial and archaeal community structure in the surface microlayer of high mountain lakes examined under two atmospheric aerosol loading scenarios. FEMS Microbiol Ecol. 2013; 84: 387–397. 10.1111/1574-6941.12068 23289422

[11] Auguet JC, Triadó-Margarit X, Nomokonova N, Camarero L, Casamayor EO. Vertical segregation and phylogenetic characterization of ammonia-oxidizing archaea in a deep oligotrophic lake. ISME J. 2012; 6: 1786–1797 10.1038/ismej.2012.33 22495069 PMC3425235

[12] Cunliffe M, Engel A, Frka S, Gašparović B, Guitart C, Murrell JC, et al Sea surface microlayers: A unified physicochemical and biological perspective of the air-ocean interface. Prog Oceanogr. 2013; 109: 104–116.

[13] Reinthaler T, Sintes E, Herndl GJ. Dissolved organic matter and bacterial production and respiration in the sea-surface microlayer of the open Atlantic and the western Mediterranean Sea. Limnol Oceanogr. 2008, 53: 122–136.

[14] Harvey GW. Microlayer collection from the surface of the sea. Limnol Oceanogr. 1966; 10: 602–605.

[15] Kutznetsova M, Lee C, Aller J. Enrichment of amino acids in the sea surface microlayer at coastal and open ocean sites in the North Atlantic Ocean. Limnol Oceanogr. 2004; 49(5): 1605–1619.

[16] Bureekul S, Murashima Y, Furutani H, Uematsu M. Enrichment of particulate phosphorus in a sea-surface microlayer over the Eastern Equatorial Pacific Ocean. Geochem J. 2012; 48(3): e1–e7.

[17] Stolle C, Labrenz M, Meeske C, Jürgens K. Bacterioneuston community structure in the Southern Baltic Sea and its dependence on meteorological conditions. Appl Environ Microbiol. 2011; 77: 3726–3733. 10.1128/AEM.00042-11 21478321 PMC3127628

[18] Wurl O, Holmes M. The gelatinous nature of the sea-surface microlayer. Mar Chem. 2008; 110: 89–97.

[19] Alldredge AL, Passow U, Logan BE. The abundance and significance of a class of large, transparent organic particles in the ocean. Deep-Sea Res Pt. I. 1993; 40: 1131–1140.

[20] Passow U, Alldredge AL. A dye-binding assay for the spectrophotometric measurement of transparent exopolymer particles (TEP). Limnol Oceanogr. 1995; 40: 1326–1335.

[21] Suzuki R, Ishimaru T. An improved method for the determination of phytoplankton chlorophyll using N,N-dimethylformamide. J Oceanogr Soc Japan. 1990; 46: 190–194.

[22] Wurl O, Miller L, Vagle S. Production and fate of transparent exopolymer particles in the ocean. J Geophys Res. 2011; 116: C00H13.

[23] Massana R, Taylor LT, Murray AE, Wu KY, Jeffrey WH, DeLong EF. Vertical distribution and temporal variation of marine planktonic archaea in the Gerlache Strait, Antartica, during early spring. Limol Oceanogr. 1998; 43: 607–617.

[24] Reysenbach A-L, Longnecker K, Kirshstein J. Novel bacterial and archaeal lineages from an in-situ growth chamber deployed at a Mid-Atlantic Ridge hydrothermal vent. Appl Environ Microbiol. 2000; 66: 3798–3806. 10966393 10.1128/aem.66.9.3798-3806.2000 PMC92223

[25] Schloss PD, Westcott SL, Ryabin T, Hall JR, Hartmann M, Hollister EB, et al Introducing mothur: Open-Source, Platform-Independent, Community-Supported Software for Describing and Comparing Microbial Communities. Appl Environ Microbiol. 2009; 75: 7537–7541. 10.1128/AEM.01541-09 19801464 PMC2786419

[26] Schloss PD, Westcott SL. Assessing and Improving Methods Used in Operational Taxonomic Unit-Based Approaches for 16S rRNA Gene Sequence Analysis. Appl Environ Microbiol. 2011; 77: 3219–3226. 10.1128/AEM.02810-10 21421784 PMC3126452

[27] Hornek R, Pommerening-Röser A, Koops H-P, Farnleitner AH, Kreuzinger N, Kirschner A, et al Primers containing universal bases reduce multiple *amoA* gene specific DGGE band patterns when analysing the diversity of beta-ammonia oxidizers in the environment. J Microbiol Methods. 2006; 66: 147–155. 10.1016/j.mimet.2005.11.001 16343671

[28] Purkhold U, Pommerening-Röser A, Juretschko S, Schmid MC, Koops H-P, Wagner M. Phylogeny of all recognized species of ammonia oxidizers based on comparative 16S rRNA and amoA sequence analysis: implications for molecular diversity surveys. Appl Environ Microbiol. 2000; 66: 5368–5382. 11097916 10.1128/aem.66.12.5368-5382.2000 PMC92470

[29] Mincer TJ, Church MJ, Taylor LT, Preston C, Kar DM, DeLong EF. Quantitative distribution of presumptive archaeal and bacterial nitrifiers in Monterey Bay and the North Pacific Subtropical Gyre. Environ Microbiol. 2007; 9: 1162–1175. 10.1111/j.1462-2920.2007.01239.x 17472632

[30] Tamura K, Stecher G, Peterson D, Filipski A, Kumar S. MEGA6: molecular evolutionary genetics analysis version 6.0. Mol Biol Evol. 2013; 30: 2725–2729. 10.1093/molbev/mst197 24132122 PMC3840312

[31] Huang Y, Niu B, Gao Y, Fu L, Li W. CD-HIT Suite: a web server for clustering and comparing biological sequences. Bioinformatics. 2010; 26: 680–682. 10.1093/bioinformatics/btq003 20053844 PMC2828112

[32] Benson DA, Clark K, Karsch-Mizrachi I, Lipman DJ, Ostell J, Sayers EW. GenBank. Nucleic Acids Res. 2015;43(Database issue):D30–D35. 10.1093/nar/gku1216 25414350 PMC4383990

[33] Tamura K, Nei M (1993) Estimation of the number of nucleotide substitutions in the control region of mitochondrial DNA in humans and chimpanzees. Mol Biol Evol 10: 512–526. 10.1093/oxfordjournals.molbev.a040023 8336541

[34] R Core Development Team. *R*: *a language and environment for statistical computing* (version 3.2.3). Vienna: R Foundation for Statistical Computing, 2015.

[35] McMurdie PJ, Holmes S. phyloseq: An R package for reproducible interactive analysis and graphics of microbiome census data. PLoS ONE. 2013; 8(4): e661217. 10.1371/journal.pone.0061217 PMC3632530 23630581

[36] Sheneman L, Evans J, Foster JA. Clearcut: a fast implementation of relaxed neighbor joining. Bioinformatics. 2006; 22(22): 2823–2824. 10.1093/bioinformatics/btl478 16982706

[37] Yamaguchi M. Growth and reproductive cycles of marine fouling ascidians *Ciona intestinalis*, *Styela plicata*, *Botrylloides violaceus* and *Leptoclinum mitsukuri* at Aburatsubo-Moroiso Inlet (Central Japan). Mar Biol. 1975; 29: 253–259.

[38] Takayanagi K, Yamada H. Effects of Benthic flux on short term variations of nutrients in Aburatsubo Bay. J Oceanogr. 1999; 463–469.

[39] Francis CA, Roberts KJ, Beman JM, Santoro AE, Oakley BB. Ubiquity and diversity of ammonia-oxidizing archaea in water columns and sediments of the ocean. Proc Natl Acad Sci USA. 2005; 102: 14683–14688. 10.1073/pnas.0506625102 16186488 PMC1253578

[40] Jung M–Y, Park S–J, Kim S–J, Kim J–G, Damsté JSS, Jeon CO, Rhee S–K. A mesophilic, autotrophic, ammonia-oxidizing archaeon of Thaumarchaeotal Group I.1a cultivated from a deep oligotrophic soil horizon. Appl Environ Microbiol. 2014; 80(12): 3465–3655. 10.1128/AEM.03730-13 PMC4054128 24705324

[41] Lebedeva EV, Hatzenpichler R, Pelletier E, Schuster N, Hauzmayer S, Bulaev A, et al Enrichment and Genome Sequence of the Group I.1a Ammonia-Oxidizing Archaeon “*Ca*. Nitrosotenuis uzonensis” Representing a Clade Globally Distributed in Thermal Habitats. PLoS ONE. 2013; 8(11): e80835 10.1371/journal.pone.0080835 24278328 PMC3835317

[42] Zhang Q, Tang F, Zhou Y, Xu J, Chen H, Wang M, Laanbroek HJ. Shifts in the pelagic ammonia-oxidizing microbial communities along the eutrophic estuary of Yong River in Ningbo City, China. Front Microbiol. 2015; 6: 1180 10.3389/fmicb.2015.01180 26579089 PMC4621301

[43] Beman JM, Popp BN, Francis CA. Molecular and biogeochemical evidence for ammonia oxidation by marine Crenarchaeota in the Gulf of California. ISME J. 2008; 2: 429–441. 10.1038/ismej.2007.118 18200070

[44] Wietz M, Gram L, Jørgensen B, Schramm A. Latitudinal patterns in the abundance of major marine bacterioplankton groups. Aquat Microbiol Ecol. 2010; 61: 179–189.

[45] Smith JM, Casciotti KL, Chavez FP, Francis CA. Differential contributions of archaeal ammonia oxidizer ecotypes to nitrification in coastal surface waters. ISME J. 2014; 8(8): 1704–1714. 10.1038/ismej.2014.11 24553472 PMC4817602

[46] Agogué H, Brink M, Dinasquet J, Herndl GJ. Major gradients in putatively nitrifying and non-nitrifying Archaea in the deep North Atlantic. Nature. 2008; 456: 788–792. 10.1038/nature07535 19037244

[47] Church MJ, Wai B, Karl DM, DeLong EF. Abundances of crenarchaeal amoA genes and transcripts in the Pacific Ocean. Environ Microbiol. 2010; 12: 679–688. 10.1111/j.1462-2920.2009.02108.x 20002133 PMC2847202

[48] Pedneault E, Galand PE, Potvin M, Tremblay J-E, Lovejoy C. Archaeal amoA and ureC genes and their transcriptional activity in the Arctic Ocean. Sci Rep. 2014; 4:4661 10.1038/srep04661 24722490 PMC3983602

[49] Hallam SJ, Konstantinos TK, Putnam N, Schelper C, Watanabe Y, Sugahara J, Preston C, de la Torre J, Richardson PM, Delong EF. Genomic analysis of the uncultivated marine crenarcheote *Crenarchaeum symbiosium*. Proc Natl Acad Sci USA. 2006; 103(48): 18296–18301. 10.1073/pnas.0608549103 17114289 PMC1643844

[50] de Corte D, Yokokawa T, Varela MM, Agogué H, Herndl GJ. Spatial distribution of bacteria and archaea and amoA gene copy numbers throughout the water column of the Eastern Mediterranean Sea. ISME J. 2008; 3(2): 147–158. 10.1038/ismej.2008.94 18818711

[51] Konstantinidis KT, Braff J, Karl DM, DeLong E. Comparative metagenome analysis of a microbial community residing at a depth of 4,000 meters at Station ALOHA in the North Pacific Subtropical Gyre. Appl Environ Microbiol. 2009; 75(16): 5345–5355. 10.1128/AEM.00473-09 19542347 PMC2725473

[52] Sintes E, Bergauer K, Corte DD, Yokokawa T, Herndl GJ. Archeal amoA gene diversity points to distinct biogeography of ammonia-oxidizing Crenarchaeota in the ocean. Environ Microbiol. 2012; 15(5): 1647–1658. 10.1111/j.1462-2920.2012.02801.x 22690844 PMC3712475

[53] Sintes E, Corte DD, Haberleitner E, Herndl GJ. Geographic distribution of archaeal ammonia oxidizing ecotypes in the Atlantic Ocean. Front Microbiol. 2016; 7: 77 10.3389/fmicb.2016.00077 26903961 PMC4746290

[54] Kalanetra KM, Bano N, Hollibaugh JT. Ammonia-oxidizing archaea in the Arctic Ocean and Antarctic coastal waters. Environ Microbiol. 2009; 11(9): 2434–2445. 10.1111/j.1462-2920.2009.01974.x 19601959

[55] Herndl GJ, Reinthaler T, Teira E, van Aken H, Veth C, Pernthaler A et al. Contribution of Archaea to total prokaryotic production in the deep Atlantic Ocean. Appl Environ Microbiol. 2005; 71: 2303–2309. 10.1128/AEM.71.5.2303-2309.2005 15870315 PMC1087563

[56] Teira E, Lebaron P, van Aken H, Herndl GJ. Distribution and activity of Bacteria and Archaea in the deep water masses of the North Atlantic. Limnol Oceanogr. 2006; 51: 2131–2144.

[57] Walker CB, de la Torre JR, Klotz MG, Urakawa H, Pinela N, Arp DJ et al *Nitrosopumilus maritimus* genome reveals unique mechanisms for nitrification and autotrophy in globally distributed marine crenarchaea. Proc Natl Acad Sci USA. 2010; 107: 8818–8823. 10.1073/pnas.0913533107 20421470 PMC2889351

[58] Massana R, Murray AE, Preston CM, DeLong EF. Vertical distributiona nd phylogenetic chracterization of marine planktonic Archaea in the Santa Barbara Channel. Appl Environ Microbiol. 1997; 63: 50–56. 8979338 10.1128/aem.63.1.50-56.1997 PMC168301

[59] Church MJ, DeLong EF, Ducklow HW, Karner MB, Preston CM, Karl DM. Abundance and distribution of planktonic Archaea and Bacteria in the waters west of the Antarctica Peninsula. Limnol Oceanogr. 2003; 48(5): 1893–1902.

[60] Luo H, Tolar BB, Swan BK, Zhang CL, Stepanauskas R, Moran MA, Hollibaugh JT. Single-cell genomics shedding light on marine Thaumarchaeota diversification. ISME J. 2014; 8(3): 732–736. 10.1038/ismej.2013.202 24196320 PMC3930325

[61] Frigaard NU, Martinez A, Mincer TJ, DeLong EF. Proteorhodopsin lateral gene transfer between marine planktonic Bacteria and Archaea. Nature. 2006; 439: 847–850. 10.1038/nature04435 16482157

[62] Herfort L, Schouten S, Abbas B, Veldhuis MJW, Coolen MJL, Wuchter C, Boon JP, Herndl GJ, Damsté JSS. Variations in spatial and temporal distribution of Archaea in the North Sea in relation to environmental variables. FEMS Microbiol Ecol. 2007; 62: 242–257. 10.1111/j.1574-6941.2007.00397.x 17991018

[63] Hugoni M, Taib N, Debroas D, Domaizon I, Dufournel IJ, Bronner G, Agogué H, Mary I, Galand PE. Structure of the rare archaeal biosphere and seasonal dynamics of active ecotypes in surface coastal waters. Proc. Natl. Acad. Sci. U.S.A. 2013; 110: 6004–6009. 10.1073/pnas.1216863110 23536290 PMC3625260

[64] Murray AE, Blakis A, Massana R, Strawzewski S, Passow A, Alldredge A, DeLong EF. A time series assessment of planktonic archaeal variability in the Santa Barbara Channel. Aquat Microb Ecol. 1999; 20: 129–145.

[65] Baier RE. “Applied chemistry at protein interfaces”, in: *Applied Chemistry at Protein Interfaces*, ed. Gould R. F., (Washington D.C: American Chemical Society); 1975 pp. 1–25.

[66] Kjelleberg S. “Mechanisms of bacterial adhesion at gas-liquid interfaces” in *Bacterial Adhesion*, eds. Savage D. C. and Fletcher M. (New York, NY: Plenum Press); 1985 pp. 163–194.

[67] Smith JM, Chavez FP, Francis CA. Ammonium uptake by phytoplankton regulates nitrification in the sunlit ocean. PLoS ONE, 2014; e108173 10.1371/journal.pone.0108173 25251022 PMC4177112

[68] Christman GD, Cottrell MT, Popp BN, Gier E, Kirchman DL. Abundance, diversity and activity of ammonia-oxidizing prokaryotes in the coastal Arctic Ocean in summer and winter. Appl Environ Microbiol. 2011; 77(6): 2016–2034. 10.1128/AEM.01907-10 PMC3067342 21239542

[69] Urakawa H, Tajima Y, Numata Y, Tsuneda S. Low temperature decreases the phylogenetic diversity of ammonia-oxidizing archaea and bacteria in aquarium biofiltration systems. Appl Environ Microbiol. 2008; 74: 894–900. 10.1128/AEM.01529-07 18065610 PMC2227712

[70] Ijichi M, Hamasaki K. Community structure of ammonia-oxidizing marine archaea differs by depth of collection and temperature of cultivation. J Oceanogr. 2011; 68: 369–386.

[71] Urakawa H, Martens-Habbena W, Huguet C, de la Torre JR, Ingalls AE, Devol AH, Stahl DA. Ammonia availability shapes the seasonal distribution and activity of archaeal and bacterial ammonia oxidizers in the Puget Sound Estuary. Limnol Oceanogr. 2014; 59: 1321–1335.

[72] Beman JM, Chow CE, King AL, Feng Y, Fuhrman JA, Andersson A, et al Global declines in oceanic nitrification rates as a consequence of ocean acidification. Proc Natl Acad Sci U.S.A. 2011; 108: 208–213. 10.1073/pnas.1011053108 21173255 PMC3017153

