# Supplementary material for: Ammonia oxidizers in the sea-surface microlayer of a coastal marine inlet
Source: PLoS One. 2018 Aug 20;13(8):e0202636. doi: 10.1371/journal.pone.0202636 (PMC6101417; doi:10.1371/journal.pone.0202636)
Supplement: S1 Table — (DOCX) [file pone.0202636.s001.docx]

**S1 Table.** Non-parametric Spearman’s rho and Kendall’s Tau correlation tests between the archaeal ammonia monooxygenase subunit A (*amoA*) gene and Marine Group-I (MG-I) 16S rRNA gene copy numbers relative to chlorophyll-a (Chl-a) and transparent exopolymer (TEP) concentrations.

|  |  | **Spearman’s Rho coefficient** | | | |
| --- | --- | --- | --- | --- | --- |
|  |  | *amoA* gene copy number | MG-I gene copy number | Chl-a concentration | TEP concentration |
| **Spearman’s Rho coefficient** | *amoA* gene copy number |  |  |  |  |
|  | MG-I 16S rRNA gene copy number | ρ = 0.93  p < 0.001 |  |  |  |
|  | Chl-a concentration | ρ = −0.88  p < 0.001 | ρ = −0.84  p < 0.001 |  |  |
|  | TEP concentration | ρ = −0.83  p < 0.001 | ρ = −0.76  p < 0.01 | ρ = 0.87  p < 0.001 |  |
