# Supplementary figures and images for: Ammonia oxidizers in the sea-surface microlayer of a coastal marine inlet
Source: PLoS One. 2018 Aug 20;13(8):e0202636. doi: 10.1371/journal.pone.0202636 (PMC6101417; doi:10.1371/journal.pone.0202636)

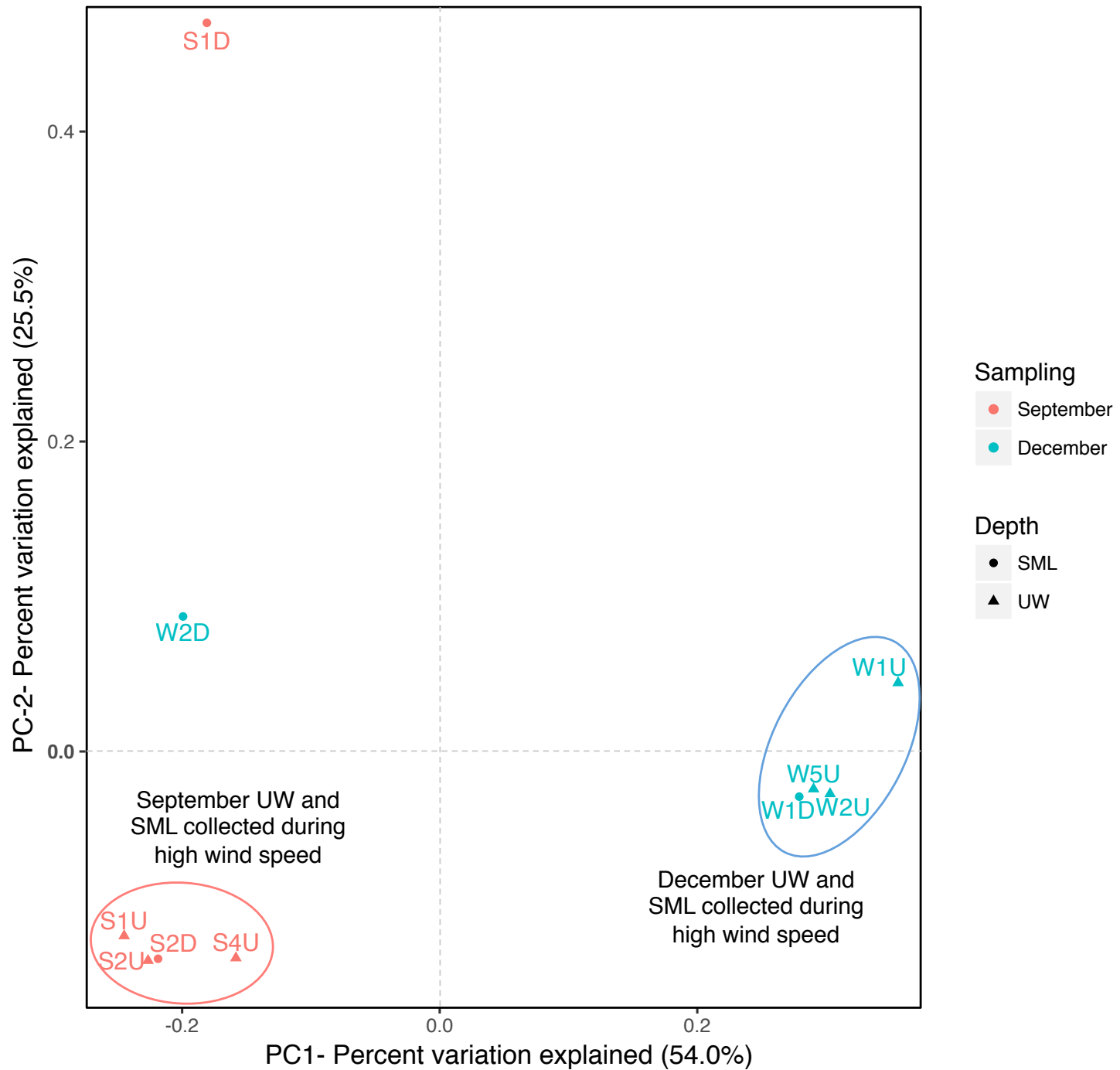

Supplement: S1 Fig — (PDF) [file pone.0202636.s002.pdf]

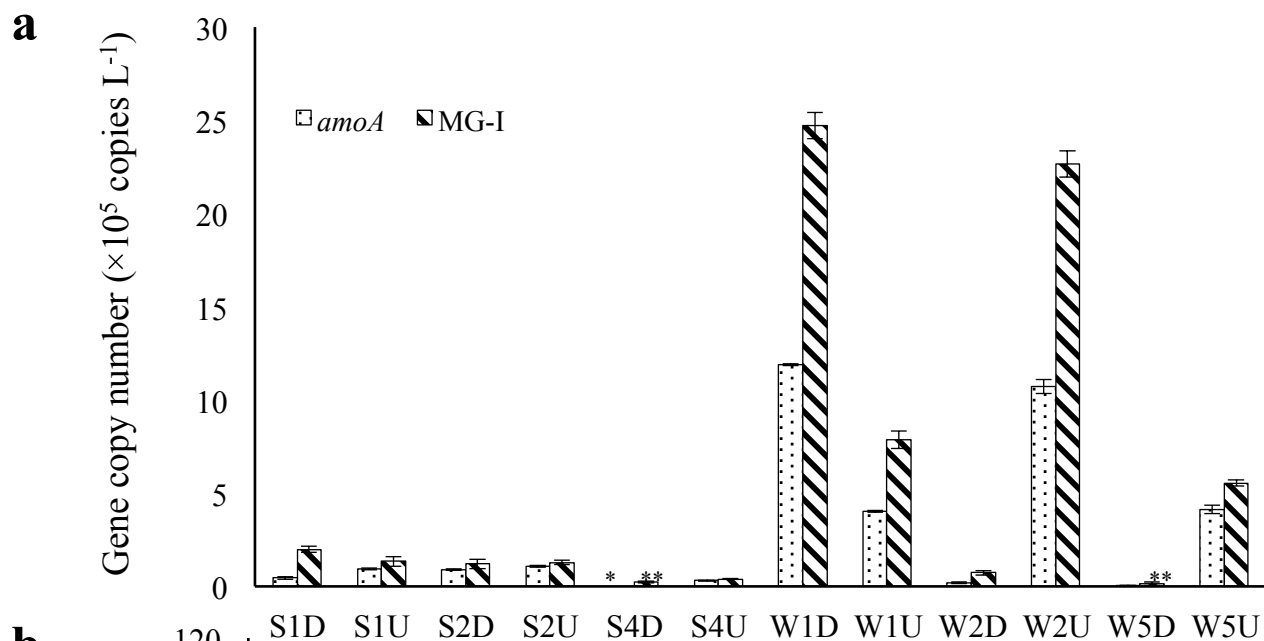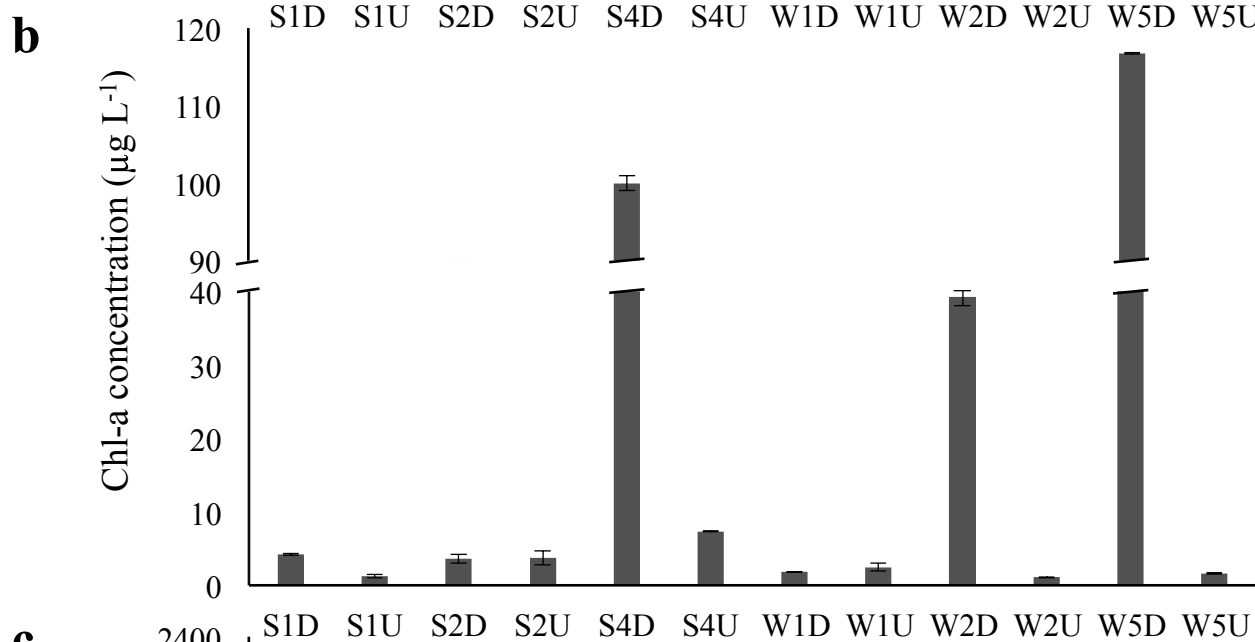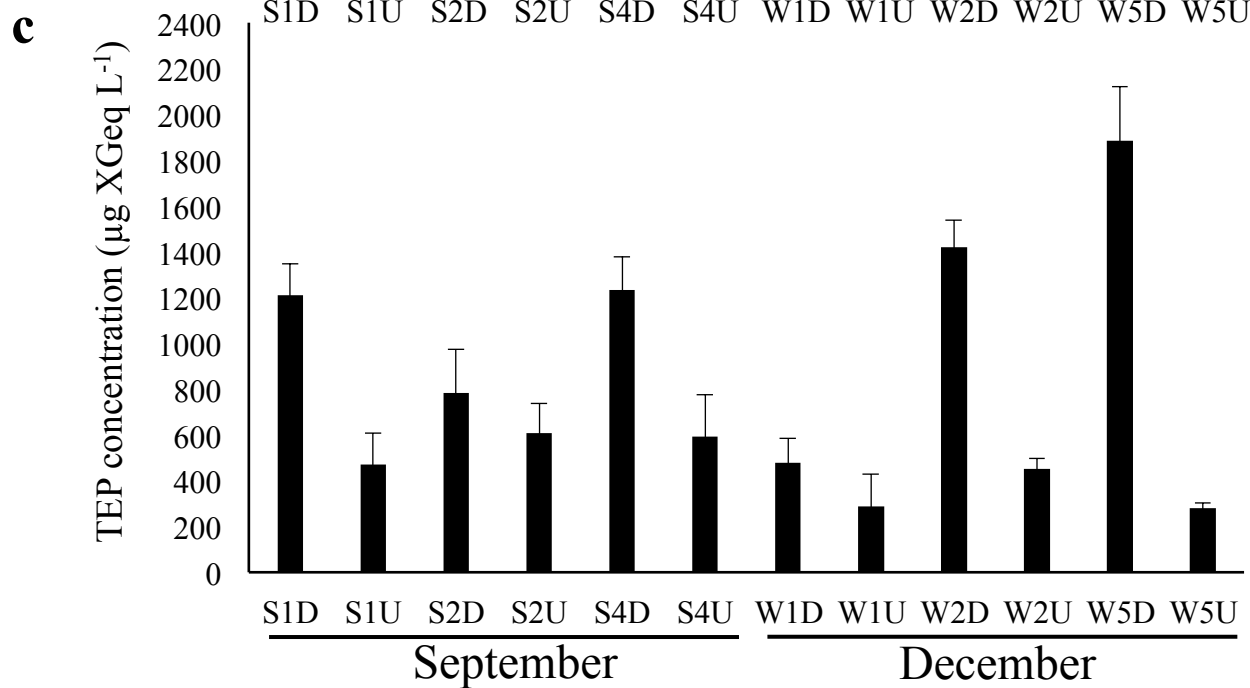

Supplement: S2 Fig — a) Copy numbers of archaeal ammonia monooxygenase subunit A (amoA) gene and archaeal Marine Group-I (MG-I) 16S rRNA gene; b) chlorophyll-a (Chl-a) and c) transparent exopolymer particles (TEP) concentrations from surface microlayer (D) and underlying water (U) samples collected in September (S) and December (W). * indicates that the concentration was below the qPCR detection limit and ** indicates that the sample was not amplified successfully using the A20F/519R primer set for 454 pyrosequencing. Error bars represent standard deviations of technical triplicates. (PDF) [file pone.0202636.s003.pdf]
